# Working Without Faces: Job Demands, Resources, and Burnout in Anonymous Helpline Workers in Slovakia and the Czech Republic—A Cross-Sectional Study

**DOI:** 10.3390/healthcare14121680

**Published:** 2026-06-12

**Authors:** Radka Čopková

**Affiliations:** Department of Economics, Faculty of Economics, Technical University of Košice, Němcovej 32, 042 00 Košice, Slovakia; radka.copkova@tuke.sk

**Keywords:** helpline workers, burnout, Job Demands–Resources model, mental health services, service-related demands, organizational support

## Abstract

Background/Objectives: Helplines are an important component of mental health support systems; however, limited research has examined burnout among helpline workers. This exploratory pilot study investigated the relationships between job demands, job and personal resources, and burnout within the Job Demands–Resources (JD-R) framework. Methods: A cross-sectional online study was conducted among 73 helpline workers. Burnout was assessed using the Burnout Assessment Tool (BAT). Job demands were categorized as topic-related, client-related, and service-related. Data were analysed using Spearman correlations and hierarchical regression analyses. Results: Job demands were positively associated with burnout (r_s_ = 0.41, *p* < 0.001), while job resources were negatively associated with burnout (r_s_ = −0.30, *p* < 0.01). Regression analyses showed that job demands and resources explained 50% of the variance in overall burnout (R^2^ = 0.50, *p* < 0.001). Service-related demands emerged as the strongest predictor of burnout (β = 0.70, *p* < 0.001) and consistently predicted exhaustion (β = 0.60, *p* < 0.001), mental distance (β = 0.48, *p* < 0.001), cognitive impairment (β = 0.52, *p* < 0.001), and emotional impairment (β = 0.61, *p* < 0.001). Organizational resources were negatively associated with mental distance (β = −0.31, *p* < 0.01), whereas topic-related demands were not significant predictors. Conclusions: The findings highlight the importance of differentiating types of job demands in understanding burnout among helpline workers. Service-related demands appeared to be more strongly associated with burnout than topic- or client-related demands, suggesting that structural aspects of helpline work may be particularly relevant for worker well-being.

## 1. Introduction

Helplines represent a form of remote support that plays an increasingly important role in health and social care systems, particularly in mental health support, prevention, and early intervention [1,2,3]. They provide immediate, accessible, and low-threshold support for individuals experiencing psychological distress, crisis, or social difficulties, often serving as a first point of contact with professional care [4,5,6]. Within healthcare systems, helplines are frequently described as “invisible healthcare providers” that bridge health, social, and community-based services [1].

The effectiveness and sustainability of helpline services depend on the well-being of their workforce. Psychological strain and burnout among helpline workers may affect not only individual functioning but also the quality, accessibility, and continuity of care provided to vulnerable populations. Helplines also reflect increasing demands on healthcare systems to provide accessible, flexible, and cost-effective support, particularly for individuals who do not seek or cannot access traditional services [1,7]. Their core functions include emotional support, crisis intervention, information provision, and referral [8,9].

A key characteristic of helplines is the provision of a safe, confidential, and often anonymous environment that facilitates disclosure of sensitive issues, especially among stigmatized or hard-to-reach populations [10,11]. As high-reach, low-cost services, helplines expand access to support and reduce inequalities in care [4,12]. They include crisis lines, general emotional support services, and specialized helplines targeting specific populations or issues [2].

### 1.1. Communication Modalities in Helpline Services

Helpline services have undergone substantial technological transformation. Traditional telephone-based support is increasingly complemented or replaced by online chat, email, and text messaging, allowing clients to choose communication channels that best match their preferences and needs [1,13].

Telephone communication enables synchronous interaction and provides paralinguistic cues, such as intonation and emotional tone, that facilitate empathic responding [14]. In contrast, text-based modalities, particularly chat, lack auditory and visual cues, which may complicate emotional interpretation and increase the risk of misunderstandings [15]. At the same time, they offer greater anonymity and control over self-presentation, which may increase clients’ willingness to disclose sensitive issues [13,16].

Email counselling is an asynchronous modality characterized by temporal flexibility and the ability to edit text, allowing clients to reflect on their concerns and workers to formulate more considered responses [14,17]. Research suggests that, particularly in child helpline services, both telephone and chat contacts contribute to improvements in well-being and reductions in perceived problem burden [17,18,19].

Overall, helplines increasingly function as multichannel systems in which communication modalities serve complementary rather than competing roles, differing in anonymity, response speed, emotional cues, and suitability for different clients and presenting problems [20].

### 1.2. Helpline Research and the Worker Perspective

Research on helpline services has predominantly focused on intervention effectiveness and factors influencing clients’ help-seeking behaviour, including reductions in psychological distress, suicidality, and perceived burden, as well as barriers and facilitators of service use [12]. In contrast, considerably less attention has been paid to helpline workers and their working conditions, despite the importance of worker well-being for service quality, user safety, and the functioning of low-threshold mental health support systems.

In many countries, helpline services are largely staffed by volunteers who complement formal health and social care systems [3,21,22]. Although trained and supervised, their engagement is primarily motivated by altruism, which has implications for workload, work organization, and service sustainability. In Slovakia and the Czech Republic, however, helpline work is more commonly performed as paid professional employment, requiring a different perspective on working conditions, job demands, and resources.

Despite evidence of psychological strain, emotional demands, and the risk of burnout among helpline workers [23,24], research in this area remains limited and fragmented [21]. The present study addresses this gap by focusing on helpline workers as key actors in service provision while considering the specific characteristics of professional helpline work in Central Europe.

### 1.3. Characteristics and Competencies of Helpline Workers

Helpline workers (also referred to as crisis line workers) provide remote psychological support through emotional support, crisis intervention, and facilitation of self-disclosure. Due to regular contact with individuals experiencing challenging life situations, this work involves specific personal and professional demands [21,25].

Research highlights the importance of empathy, warmth, patience, genuineness, emotional stability, and a non-judgmental attitude in helpline work [2,26]. Workers are also expected to demonstrate emotional resilience and the ability to respond sensitively to clients’ distress [21,27].

These characteristics are closely linked to communication and counselling competencies, including active listening, emotional reflection, paraphrasing, and the use of open-ended questions [28]. Such skills are essential in short-term and crisis-oriented interactions, where workers primarily support coping, emotional validation, and client autonomy rather than providing psychotherapy in a strict sense [13,22].

Helpline workers must also rapidly assess clients’ situations, identify risk factors such as suicidality, and provide appropriate referrals [25]. Professional preparation, therefore, typically includes specialized training, supervision, and the development of coping strategies for managing long-term emotional demands [22].

### 1.4. Factors Influencing the Well-Being of Helpline Workers

The well-being of helpline workers is shaped by several interrelated factors that can be categorized into three domains: (1) nature of work, (2) organization-related factors, and (3) worker-related factors [3,21,22]. This framework also served as a conceptual basis for operationalizing job demands and resources in the present study.

#### 1.4.1. Nature of Work

The nature of helpline work reflects daily interactions between workers and clients and includes structural, interactional, and temporal demands. A defining feature is anonymity, which facilitates disclosure of sensitive issues [16] while increasing uncertainty, unpredictability, and psychological strain for workers [29]. Anonymity limits the ability to verify clients’ situations and may enable inappropriate or abusive interactions [21,25]. At the same time, it may create emotional distance while increasing feelings of reduced control over outcomes [22,30].

Workers are frequently exposed to challenging client behaviours, including manipulative, uncooperative, intoxicated, aggressive, or repeated contacts, as well as boundary violations [21,31,32]. Particularly demanding are “difficult calls”, such as silent, aggressive, or obscene interactions [2,21]. Emotional strain is further increased by frequent exposure to suicidality, violence, abuse, self-harm, and severe psychological distress, with suicidality perceived as especially demanding [22,24].

Temporal and workload-related demands also contribute to strain. Helpline contacts are often one-time, time-limited, and urgent, requiring rapid assessment and intervention without the possibility of long-term engagement [3,25]. Workload reflects not only the number of contacts but also their emotional intensity and the need for sustained attention during demanding shifts [21,33,34].

Additional challenges arise from the absence of non-verbal cues, particularly in text-based communication, which complicates emotional interpretation and increases uncertainty regarding the client’s situation [22,28,35]. This uncertainty may be amplified by technical disruptions, delayed responses, and the lack of follow-up in one-off interactions [36,37,38].

Helpline work also requires managing professional boundaries in emotionally intense, short-term, and anonymous interactions, balancing empathy with professional distance [25,39]. These demands are closely linked to ethical responsibilities, including safe intervention and balancing client anonymity with the duty to act in situations of risk [40,41].

Finally, workload and job control play an important role in worker well-being. High cumulative workload, driven by contact frequency and shift duration, is associated with increased burnout risk, particularly emotional exhaustion [22,34,42]. In contrast, greater job control and autonomy are associated with lower burnout and higher professional efficacy [43,44].

#### 1.4.2. Organization-Related Factors

In addition to the nature of the work, the organizational environment plays an important role in shaping helpline workers’ well-being and may serve as both a protective factor and a source of strain [3,21,22].

A central organizational factor is supervision, which provides space to reflect on challenging interactions, process emotions, and receive professional guidance. Supervision also facilitates experience sharing, validation of emotional responses, and reduces the sense of isolation associated with remote and anonymous work, while serving as a protective factor against burnout [21,24].

Training represents another important factor, as workers must be prepared for the demands of remote communication, crisis intervention, and work with diverse client populations. Training typically focuses on communication and counselling skills, as well as competencies related to managing high-risk situations, ethical dilemmas, and emotional responses. Insufficient preparation may increase uncertainty and reduce self-efficacy [2,21,28].

Team support also contributes to worker well-being. Despite the individual nature of helpline work, teams provide emotional and practical support through shared experiences, consultation, and a sense of belonging, which may reduce stress and normalize workers’ experiences [21,23,34].

Broader organizational support, including resource availability, quality of management, clarity of procedures, and work culture, also plays an important role in reducing stress and preventing burnout. Conversely, insufficient support, unclear roles, and high demands without adequate resources may negatively affect worker well-being and service quality [21,45,46].

#### 1.4.3. Worker-Related Factors

The third group of factors includes individual characteristics that shape helpline workers’ responses to work demands and may function as both risk and protective factors. These include demographic characteristics such as age, family status, and socioeconomic background. Research suggests that helpline workers, particularly volunteers, are often middle-aged, well-educated, and socially stable, which may influence both their motivation and their capacity to cope with demanding situations [2,22].

Professional background, including prior experience, education, and training, also plays an important role. More experienced workers may be better equipped to manage challenging interactions, although prolonged exposure to clients’ distress may also contribute to cumulative strain [13,22,34].

Coping strategies are another important determinant of well-being. Adaptive strategies, such as seeking social support, reflection, and maintaining work–life boundaries, are associated with protective effects, whereas maladaptive coping increases the risk of emotional exhaustion and burnout [22,47].

Empathy, as a core characteristic of helpline work, has an ambivalent role. Although it enhances the quality of support, it may also increase the risk of emotional exhaustion, particularly during repeated exposure to clients’ traumatic experiences [2,14,21].

#### 1.4.4. Burnout in Helpline Work

In addition to positive outcomes such as job satisfaction, meaning, and personal growth, prolonged exposure to the demands of helpline work may lead to burnout. Burnout is understood as a consequence of chronic work-related stress characterized by exhaustion, mental distance, and reduced professional efficacy [48]. Building on the classical framework of Maslach et al. [49], the present study adopts a multidimensional conceptualization of burnout that includes exhaustion, mental distance, cognitive impairment, and emotional impairment [50,51].

Within helping professions, burnout is among the most extensively studied negative outcomes and is associated with prolonged exposure to human suffering, high emotional demands, and responsibility for others [52,53,54,55]. Burnout may affect not only individual functioning but also quality of care, team stability, and service sustainability [43].

Research on burnout among helpline workers remains relatively limited and fragmented [2,3,21,22]. Existing evidence nevertheless suggests that workers are exposed to a distinct combination of stressors, including suicidal calls, violence, abuse, aggressive or inappropriate client behaviour, urgency, and cumulative stress [25,56,57]. Organizational factors such as insufficient support, feedback, and training may further increase burnout risk, whereas supervision and adequate preparation appear protective [2].

Research also highlights additional psychological consequences, including positive and negative emotional responses, vicarious traumatization, and secondary traumatic stress [21,24]. Moreover, characteristics of helpline work, such as anonymity, one-off interactions, limited feedback, and decision making under uncertainty, may contribute to feelings of frustration and ineffectiveness [2].

Overall, helpline work presents a distinct configuration of job demands, underscoring the need for an appropriate theoretical framework to understand burnout in this context.

#### 1.4.5. The Job Demands–Resources Model

One of the most influential frameworks for explaining burnout is the Job Demands–Resources (JD-R) model, which conceptualizes the work environment in terms of job demands and job resources [58]. Job demands refer to aspects of work that require sustained effort and are associated with physiological or psychological costs, whereas job resources facilitate goal attainment, reduce demands, and support personal development [59]. The model distinguishes between a health impairment process, in which high demands lead to exhaustion and burnout, and a motivational process, in which resources promote engagement and well-being [58].

Empirical applications of the JD-R model have been demonstrated in call centre environments, which share structural similarities with helpline services, such as high contact frequency, time pressure, and mediated communication. However, unlike performance-oriented call centres, helpline work is characterized by greater emotional involvement and crisis-related interactions [3,21,22]. Research in these settings has identified key demands, including workload, emotional strain, and technical issues, as well as resources such as social support, supervision, feedback, and control over working time, all of which influence engagement and retention [59].

In the context of helpline services, the JD-R model provides a useful framework for organizing factors related to worker well-being. Nature-of-work factors can be conceptualized as job demands, organizational factors as job resources, and individual characteristics as personal resources that shape the relationship between demands and outcomes [58]. This alignment supports the use of the JD-R model to examine burnout among helpline workers and to understand the balance between well-being and strain in this context.

### 1.5. Present Study

Despite the growing body of research on helpline services, studies focusing on helpline workers, particularly on burnout, remain limited and fragmented. Existing knowledge is largely derived from research on helping professions or call centre environments, which provide useful insights but do not fully capture the specific characteristics of helpline work. Moreover, prior studies often examine isolated factors rather than integrating them within a unified theoretical framework that would enable a more comprehensive understanding of the relationships between job demands, resources, and burnout.

There is also a lack of empirical research addressing the specific structure of job demands in helpline contexts. Although the Job Demands–Resources model offers a robust framework, its application to helpline services remains underdeveloped, particularly in differentiating demands related to contact topics, client behaviour, and service-delivery structure. Similarly, few studies have simultaneously examined the role of organizational and personal resources within a single analytical model.

Another limitation concerns the contextual specificity of existing research, which has predominantly focused on volunteer-based helpline settings. This limits the generalizability of findings to contexts where helpline work is performed as a paid profession, where differences in job demands, resource availability, and overall work dynamics may be expected.

In response to these gaps, the present study examines burnout among helpline workers within the framework of the Job Demands–Resources model, integrating nature-of-work, organizational, and individual factors [3,21,22]. Particular emphasis is placed on differentiating job demands into topic-, client-, and service-related categories, as well as on the roles of organizational and personal resources.

The aim of the study is to examine the relationships between job demands, job and personal resources, and multiple dimensions of burnout among helpline workers. Based on the JD-R model and existing evidence, the following hypotheses were formulated:

**H1.**  *Higher levels of job demands (topic-related, client-related, and service-related) will be positively associated with burnout.*

**H2.**  *Higher levels of job resources will be negatively associated with burnout.*

**H3.**  *Job demands will be significant positive predictors of burnout.*

**H4.**  *Job resources will be significant negative predictors of burnout and will explain additional variance in burnout beyond job demands.*

## 2. Methods

### 2.1. Study Design

The study employed a cross-sectional, exploratory, pilot design, using an anonymous online survey administered via Google Forms. The questionnaire was self-administered and completed electronically, available in Slovak and Czech language versions. To ensure confidentiality and encourage open responding, no IP addresses or other identifying metadata were collected. In addition to examining relationships between job demands, job resources, and burnout among helpline workers, the study also served as an initial empirical examination of an author-developed instrument designed for the specific context of anonymous helpline work.

### 2.2. Setting

Data were collected between March and April 2026 among helpline workers employed in psychosocial support helpline services operating in Slovakia and the Czech Republic. The included services provided anonymous distance counsellingcounselling via telephone, online chat, and email.

### 2.3. Participants

Participants were recruited using a combination of direct and snowball sampling. Managers of helpline services in Slovakia and the Czech Republic were contacted via email and asked to distribute the survey link among their staff. In total, 50 helpline services were approached. This recruitment strategy was selected due to the relatively small and specialized nature of the target population. Snowball sampling is considered an appropriate and commonly used approach in research involving hard-to-reach professional groups.

Eligibility criteria required participants to be actively working paid helpline workers providing anonymous distance counselling at the time of data collection. Only individuals engaged in telephone, chat, or email counselling were included in the study.

Prior to participation, respondents were presented with an informed consent form describing the purpose of the study, voluntary participation, anonymity of responses, and the right to withdraw at any time without consequences. Completion of the questionnaire took approximately 10–15 min. No personally identifiable information was collected.

The study was conducted in accordance with the Declaration of Helsinki and approved by the Ethics Committee of the Technical University of Košice (No. 7703).

### 2.4. Variables

The primary outcome variable was burnout, conceptualized as a multidimensional construct consisting of exhaustion, mental distance, cognitive impairment, and emotional impairment.

Predictor variables included job demands and job resources. Job demands were categorized into topic-related, client-related, and service-related demands. Job resources were divided into personal resources and organizational resources.

Additional sociodemographic and work-related variables included gender, age, length of professional experience in helpline services, type of employment contract, average monthly working hours, communication modality, work setting, engagement in face-to-face counselling, and professional background.

### 2.5. Measures

Data were collected using a structured online questionnaire consisting of three main parts: (1) sociodemographic and work-related characteristics, (2) burnout, and (3) work-related factors, including job demands and job resources.

All instruments were administered in Slovak and Czech. The Slovak and Czech versions of the Burnout Assessment Tool (BAT) were used, while the author-developed questionnaire was originally created in Slovak and subsequently adapted into Czech for the present study. Although Slovak and Czech are highly mutually intelligible languages, the Czech version was reviewed by a Czech expert in helpline practice to ensure linguistic appropriateness and contextual clarity.

***Sociodemographic and Work-Related Variables***: Participants provided information on gender, age, length of experience with helpline services, type of employment contract (full-time, part-time, or casual), and average monthly working hours. Additional variables included engagement in face-to-face counselling, primary mode of service delivery (telephone, chat, email), work setting (on-site, home office, or hybrid), and professional background (e.g., psychology, social work, education, healthcare, or other).

***Burnout assessment tool*** ([50], for Slovak version see Bačkorová et al. [60]; for Czech version see Procházka et al. [61]): Burnout was measured using the Slovak version of the Burnout Assessment Tool (BAT), which conceptualizes burnout as a multidimensional construct consisting of four core components: exhaustion, mental distance, cognitive impairment, and emotional impairment.

The instrument consists of 12 items (3 items per dimension). Example items include: “I feel mentally exhausted” (exhaustion), “I feel a strong aversion toward my job” (mental distance), “I have trouble staying focused” (cognitive impairment), and “At work, I feel unable to control my emotions” (emotional impairment).

Participants rated the frequency of each experience on a 5-point Likert scale ranging from 1 (never) to 5 (always). Higher scores indicate higher levels of burnout.

In the present study, the BAT demonstrated good internal consistency (ω = 0.885), with acceptable to good reliability for all subscales (exhaustion ω = 0.769; mental distance ω = 0.765; cognitive impairment ω = 0.838; emotional impairment ω = 0.834).

***Job Demands and Job Resources***: The author-developed questionnaire was conceptually grounded in the Job Demands–Resources framework [58] and further informed by empirical findings on helpline workers’ well-being, particularly the categorization of factors into work-related, organizational, and individual domains [3,21,22]. Existing JD-R measurement approaches typically conceptualize working conditions in terms of broader categories of job demands and job resources, such as workload, emotional demands, job control, supervisory support, feedback, growth opportunities, and organizational support [59,62]. In previous JD-R studies conducted in helping professions and call-centre environments, these dimensions have primarily been operationalized as general psychosocial characteristics of the work environment.

The present instrument extends these approaches by differentiating job demands according to their specific source within anonymous helpline settings. In contrast to traditional JD-R operationalizations that focus mainly on quantitative workload, emotional demands, or organizational characteristics, the current questionnaire distinguished among topic-related, client-related, and service-related demands. This distinction was designed to capture characteristics that may be particularly salient in anonymous distance counselling but less visible in broader JD-R measures, including the anonymity of clients, uncertainty in interactions, the lack of nonverbal cues, emotionally intensive crisis communication, and demands associated with text-based counselling modalities.

The instrument’s structure was informed by existing JD-R literature, prior JD-R measurement approaches, and research on helping professions and call-centre work environments (e.g., [59,62]). At the same time, the instrument was designed to reflect the specific organizational and communication context of helpline services. The aim was not to replace existing JD-R instruments, but to provide an initial, exploratory, and context-sensitive operationalization of job demands and resources in anonymous helpline work.

Job demands were operationalized across three domains:

- **Topic-related demands** (**8 items**) capturing exposure to emotionally demanding client issues (e.g., suicidality, self-harm, abuse). Example item: “During my shift, I deal with situations in which a client expresses an immediate intention to commit suicide.”
- **Client-related demands** (**10 items**) reflecting challenging client behaviours (e.g., hostility, passivity, repeated contacts, dependency). Example item: “During my shift, I face hostile or disrespectful reactions from clients.”
- **Service-related demands** (**26 items**) representing structural and situational aspects of helpline work (e.g., absence of non-verbal cues, uncertainty, ethical dilemmas, time pressure, emotional intensity). Example item: “The absence of non-verbal cues makes it difficult for me to understand the client’s emotional experience.”

Items were rated on a 5-point scale ranging from 1 (strongly disagree) to 5 (strongly agree), reflecting perceived burden.

The overall job demands scale demonstrated excellent internal consistency (ω = 0.932), with good reliability for all subscales (topic-related ω = 0.825; client-related ω = 0.831; service-related ω = 0.896).

Job resources were assessed in two domains:

- **Personal resources** (**8 items**) referring to individual coping capacities, such as emotional regulation, detachment from work, and acceptance of intervention limits. Example item: “After finishing my shift, I am able to stop thinking about contacts with clients.”
- **Organizational resources** (**12 items**) reflecting support provided by the organization, including supervision, team support, training, autonomy, and methodological guidance. Example item: “I have access to regular supervision or peer consultation.”

All items were rated on a 5-point Likert scale ranging from 1 (strongly disagree) to 5 (strongly agree), with higher scores indicating greater perceived resources.

The overall job resources scale showed good internal consistency (ω = 0.857), with subscale reliabilities of ω = 0.826 (personal resources) and ω = 0.822 (organizational resources). In addition to internal consistency coefficients, corrected item–total correlations were examined to assess item coherence within the individual subscales. The obtained values ranged from 0.15 to 0.71 for topic-related demands, from 0.29 to 0.67 for client-related demands, from 0.13 to 0.65 for service-related demands, from 0.29 to 0.69 for personal resources, and from 0.17 to 0.65 for organizational resources. Overall, these results provided preliminary support for internal coherence at the item level, although some items demonstrated weaker associations with their respective subscales.

The questionnaire domains were directly linked to the study hypotheses and the JD-R framework. Topic-, client-, and service-related demands comprised the job demands component of the model, whereas personal and organizational resources comprised the job resources component. These variables were subsequently analysed as predictors of overall burnout and its individual dimensions.

To support content validity and clarity, the items were reviewed by experts in helpline practice. Given the exploratory pilot nature of the study, psychometric evaluation of the instrument focused primarily on internal consistency and content validity. The sample size did not allow for robust exploratory or confirmatory factor-analytic procedures.

### 2.6. Bias

Several steps were taken to reduce potential sources of bias. The survey was administered anonymously to reduce social desirability bias and encourage honest reporting of work-related experiences and burnout symptoms. Standardized online administration ensured that all participants received the same instructions and questionnaire format.

Because all variables were collected using self-report measures at a single time point, the study was potentially susceptible to common method variance. To assess this risk, Harman’s single-factor test was conducted using an unrotated exploratory factor analysis. The first unrotated factor accounted for 18.41% of the total variance, suggesting that common method bias was unlikely to substantially affect the findings.

Given the relatively small and specialized nature of the target population, participants were recruited using direct and snowball sampling. Although this approach was considered appropriate for accessing helpline workers, it may have increased the risk of self-selection bias and limited the representativeness of the sample.

Regarding missing data, some questionnaire items were not applicable to all respondents because they referred specifically to written forms of counselling (e.g., chat or email), which were not provided by all participants. These responses were treated as structurally missing rather than missing at random. Scale scores were computed using the mean of available items, provided that a sufficient proportion of items within the scale had been completed.

### 2.7. Study Size

The sample size was determined by the accessibility of the target population, as helpline workers are a relatively small, hard-to-reach professional group in Slovakia and the Czech Republic.

To evaluate the adequacy of the obtained sample, a post hoc power analysis was conducted using G*Power 3.1 for multiple linear regression models with five predictors, α = 0.05, and a total sample size of 73. Effect sizes were calculated from the observed R^2^ values using Cohen’s f^2^. The achieved statistical power ranged from approximately 0.95 to >0.99 across the regression models, indicating sufficient power to detect the observed effects.

### 2.8. Quantitative Analysis

Quantitative variables were analysed primarily as continuous variables. Scale scores for burnout, job demands, and job resources were computed as the mean scores across corresponding items, with higher scores indicating higher levels of the respective constructs.

Burnout was analysed both as a global construct and at the level of its four dimensions: exhaustion, mental distance, cognitive impairment, and emotional impairment. Job demands were examined across three domains (topic-related, client-related, and service-related), while job resources were analysed across two domains (personal and organizational).

Continuous variables such as age, professional experience, and average monthly working hours were retained in their original metric forms for all analyses. Employment type, communication modality, and work setting were treated as categorical variables and used descriptively.

### 2.9. Data Analysis

Data were analysed using Jamovi statistical software (Version 2.7.6). Descriptive statistics, including means, standard deviations, medians, skewness, and kurtosis, were computed for all study variables. The normality of distributions was further examined using the Shapiro–Wilk test. Given that several variables deviated significantly from normality, nonparametric methods were applied where appropriate. Internal consistency of all scales was assessed using McDonald’s omega (ω) coefficients. Associations between variables were examined using Spearman’s rank-order correlation coefficients, due to deviations from normality and the ordinal nature of the data.

Prior to the main analyses, assumptions for regression analysis were evaluated, including linearity, homoscedasticity, and multicollinearity. Although several variables deviated from normality according to the Shapiro–Wilk test, the assumptions of linear regression were evaluated primarily based on residual distributions rather than the normality of individual observed variables. As recommended by Tabachnick and Fidell [63] and Field [64], inspection of residual plots and Q–Q plots was used to assess normality, linearity, and homoscedasticity of residuals. The residual diagnostics did not indicate substantial violations of these assumptions. Therefore, linear regression analyses were considered appropriate for the present exploratory pilot data.

To examine the relationships between work-related factors and burnout, a series of hierarchical multiple regression analyses was conducted. Burnout was analysed both as a global construct and at the level of its four dimensions: exhaustion, mental distance, cognitive impairment, and emotional impairment.

In each regression model, predictors were entered in two steps. In the first step, job demands (topic-related, client-related, and service-related) were included. In the second step, job resources (personal and organizational) were added to assess their incremental contribution beyond job demands.

Statistical significance was evaluated at the *p* < 0.05 level.

## 3. Results

### 3.1. Participants

The sample consisted of 73 helpline workers, including 10 men (14%) and 63 women (86%). The mean age was 31.2 years for men (SD = 10.25; range = 23–52) and 34.89 years for women (SD = 12.46; range = 21–71).

Participants reported an average of 4.43 years of professional experience in helpline services (range = 0.25–23). When stratified by employment type, the mean length of practice was comparable across groups: 4.37 years for workers on casual contracts (range = 0.25–20), 4.49 years for full-time employees (range = 0.5–23), and 4.50 years for part-time employees (range = 0.3–15).

The average workload was 66.5 h per month (range = 4–180), with substantial variation across employment types. Workers on casual contracts reported an average of 26.6 h (range = 8–50), part-time workers 64.7 h (range = 4–100), and full-time employees 127.38 h per month (range = 24–180).

Participants were employed across a range of anonymous helpline services, including general and crisis helplines, as well as specialized services focusing on areas such as violence, addictions, LGBTQ+ issues, legal problems, HIV/AIDS, and oncology-related support. Some services were also tailored to specific populations (e.g., children, parents, or older adults).

All participants provided distance counselling through at least one communication modality, including telephone, online chat, or email.

The characteristics of the sample are presented in Table 1. Most respondents were employed under casual work contracts (49%), followed by full-time (33%) and part-time (18%) positions. Approximately one-third (32%) of participants reported providing face-to-face counselling in addition to helpline work.

Regarding professional background, the largest proportion of participants were trained in psychology (59%), followed by social work (30%), while 11% reported other professional orientations. Regarding work setting, most respondents worked on-site (71%), with smaller proportions working from home (15%) or in a hybrid format (14%).

Participants used multiple communication channels in their work: 20% used a single channel, 43% used two, and 37% used three (e.g., telephone, chat, and email).

### 3.2. Descriptive Statistics and Preliminary Analyses

Descriptive statistics for the main study variables are presented in Table 2. All variables were computed as mean scale scores, with higher values indicating higher levels of the respective constructs.

Overall, the mean level of burnout was relatively low (M = 1.86, SD = 0.53), with the highest scores observed for exhaustion (M = 2.38, SD = 0.74) and the lowest for emotional impairment (M = 1.53, SD = 0.63).

Job demands were reported at a moderate level (M = 2.99, SD = 0.49). Among the subdimensions, topic-related demands showed the highest mean (M = 3.25, SD = 0.81), followed by client-related demands (M = 3.13, SD = 0.75), while service-related demands were somewhat lower (M = 2.85, SD = 0.49).

In contrast, job resources were rated relatively high (M = 4.24, SD = 0.44), both for personal (M = 4.14, SD = 0.59) and organizational (M = 4.31, SD = 0.49) resources.

Examination of distributional properties indicated that several variables deviated from normality. Burnout and its subdimensions showed positive skewness and elevated kurtosis, suggesting a concentration of lower scores and a non-normal distribution. These findings were confirmed by the Shapiro–Wilk test, which was significant for most variables (*p* < 0.05).

### 3.3. Correlation Analysis

Spearman correlation coefficients between the main study variables are presented in Table 3.

Overall, burnout was positively associated with job demands (r_s_ = 0.41, *p* < 0.001), with the strongest relationship observed for service-related demands (r_s_ = 0.55, *p* < 0.001). In contrast, associations with topic-related demands (r_s_ = 0.13, ns) and client-related demands (r_s_ = 0.21, ns) were weaker and not statistically significant.

Job resources were negatively associated with burnout (r_s_ = −0.30, *p* < 0.01). At the subscale level, personal resources showed a significant negative association (r_s_ = −0.25, *p* < 0.01), whereas the association with organizational resources was weaker and not statistically significant (r_s_ = −0.20, ns).

At the level of burnout dimensions, service-related demands demonstrated consistent positive associations across all components, including exhaustion (r_s_ = 0.57, *p* < 0.001), mental distance (r_s_ = 0.27, *p* < 0.05), cognitive impairment (r_s_ = 0.23, *p* < 0.05), and emotional impairment (r_s_ = 0.44, *p* < 0.001).

Client-related demands were significantly associated with exhaustion (r_s_ = 0.27, *p* < 0.05) and mental distance (r_s_ = 0.24, *p* < 0.05), while topic-related demands showed no significant associations with burnout dimensions.

Regarding job resources, both personal (r_s_ = −0.23, *p* < 0.05) and organizational (r_s_ = −0.27, *p* < 0.05) resources were significantly associated with mental distance, whereas their associations with other burnout dimensions were weak or non-significant.

### 3.4. Hierarchical Regression Analyses

To further examine the relationships between job demands, job resources, and burnout, a series of hierarchical multiple regression analyses was conducted.

Multicollinearity diagnostics indicated no serious multicollinearity concerns. Variance inflation factors (VIF) ranged from 1.26 to 1.81, while tolerance values ranged from 0.55 to 0.79, remaining within commonly recommended thresholds for regression analyses.

Burnout was analysed both as a global construct and at the level of its four dimensions. Results are presented in Table 4, Table 5, Table 6, Table 7 and Table 8.

In Model 1, job demands explained a significant proportion of variance in burnout (R^2^ = 0.48, *p* < 0.001). Among the predictors, service-related demands emerged as a strong positive predictor (β = 0.68, *p* < 0.001), whereas topic-related and client-related demands were not significant.

In Model 2, the inclusion of job resources resulted in a small, non-significant increase in explained variance (ΔR^2^ = 0.02, ns), with the overall model remaining significant (R^2^ = 0.50, *p* < 0.001). Service-related demands remained a strong predictor (β = 0.70, *p* < 0.001), while neither personal resources (β = 0.11, ns) nor organizational resources (β = −0.13, ns) reached statistical significance.

The results for exhaustion are presented in Table 5.

In Model 1, job demands accounted for a significant proportion of variance (R^2^ = 0.37, *p* < 0.001), with service-related demands as the only significant predictor (β = 0.59, *p* < 0.001).

The addition of job resources in Model 2 did not significantly improve the model (ΔR^2^ = 0.00), and neither personal nor organizational resources were significant predictors.

The regression analysis for mental distance is presented in Table 6.

In Model 1, job demands explained 29% of the variance (R^2^ = 0.29, *p* < 0.001). Both client-related demands (β = 0.34, *p* < 0.05) and service-related demands (β = 0.43, *p* < 0.001) were significant positive predictors, while topic-related demands showed a negative association (β = −0.30, *p* < 0.05).

In Model 2, the inclusion of job resources led to a significant increase in explained variance (ΔR^2^ = 0.09, *p* < 0.05), with the total model explaining 38% of the variance (R^2^ = 0.38, *p* < 0.001). In this model, service-related demands remained a significant positive predictor (β = 0.48, *p* < 0.001), and client-related demands also retained significance (β = 0.27, *p* < 0.05).

Importantly, both personal resources (β = 0.26, *p* < 0.05) and organizational resources (β = −0.31, *p* < 0.01) emerged as significant predictors, but with opposite directions of association.

The results for cognitive impairment are presented in Table 7.

In Model 1, job demands explained 23% of the variance (R^2^ = 0.23, *p* < 0.001), with service-related demands as the only significant predictor (β = 0.52, *p* < 0.001).

Adding job resources did not improve the model (ΔR^2^ = 0.00), and no additional predictors reached statistical significance.

The regression model for emotional impairment is presented in Table 8.

In Model 1, job demands explained a significant proportion of variance (R^2^ = 0.36, *p* < 0.001), with service-related demands as the only significant predictor (β = 0.60, *p* < 0.001).

In Model 2, the inclusion of job resources did not significantly increase the explained variance (ΔR^2^ = 0.00), and neither personal nor organizational resources were significant predictors.

Across all models, service-related demands consistently emerged as the strongest and most robust predictor of burnout and its dimensions. Topic-related demands showed no significant effects, while client-related demands were relevant only for mental distance.

Job resources demonstrated a limited, dimension-specific role, contributing significantly only to mental distance, where organizational resources were negatively associated, and personal resources were positively associated with this dimension.

The analyses provided partial support for the proposed hypotheses.

Hypothesis 1, which predicted that job demands would be positively associated with burnout, was partially supported. While overall job demands were positively related to burnout, only service-related demands consistently emerged as a significant positive predictor across all regression models. Topic-related demands were not significant predictors, and client-related demands showed limited effects, being associated only with mental distance.

Hypothesis 2, which assumed that job resources would be negatively associated with burnout, was also partially supported. Job resources were negatively correlated with burnout; however, their predictive role in regression analyses was limited. A significant effect was observed only for mental distance, where organizational resources were negatively associated, while personal resources showed a positive association.

Hypothesis 3, stating that job demands would remain significant predictors of burnout, was supported, as service-related demands consistently predicted burnout and its dimensions across all models.

Hypothesis 4, which proposed that job resources would contribute to explaining burnout beyond job demands, was only partially supported. Job resources did not significantly increase explained variance in most models, except for mental distance, where their inclusion led to a significant improvement in model fit.

## 4. Discussion

The present study examined the relationships among job demands, job resources, and burnout among helpline workers within the Job Demands–Resources (JD-R) framework, with particular emphasis on distinguishing types of job demands specific to helpline work. Importantly, the study focused on anonymous helpline services, representing a distinct context within helping professions.

From a healthcare perspective, these findings are highly relevant, as helplines constitute an essential component of contemporary mental health systems, particularly in prevention, early intervention, and crisis support. The well-being of helpline workers, therefore, represents not only an individual or organizational concern but also a system-level factor influencing the quality and sustainability of care.

Anonymity fundamentally shapes helpline interactions. While it facilitates disclosure and access to support, it also introduces uncertainty, limited feedback, reduced control over outcomes, and increased ambiguity, which represent key sources of strain [2,3,21,22,29,30]. By focusing on anonymous services, the study captures a specific configuration of job demands that is not fully represented in other helping professions or in non-anonymous contexts.

Addressing an identified research gap, the study provides an integrated and context-sensitive examination of burnout by simultaneously considering topic-, client-, and service-related demands, alongside personal and organizational resources. Previous research has typically focused on isolated factors, failing to capture the specific configuration of stressors inherent to helpline work [3,12,21,22].

By differentiating these domains, the findings extend the JD-R model and offer a more nuanced understanding of how service structural characteristics contribute to burnout in this context.

The most salient finding of this study is the consistent and dominant role of service-related demands as predictors of burnout across all dimensions. From a healthcare perspective, this highlights the importance of structural characteristics of service delivery, as these factors directly influence both worker well-being and the capacity of services to respond to increasing mental health needs. While job demands as a general category were associated with burnout, only service-related demands demonstrated robust and stable effects.

This finding aligns with previous research emphasizing the role of structural characteristics of helpline work—such as time pressure, uncertainty, emotional intensity, and absence of non-verbal cues—as key sources of strain [3,21,22,33,34] and supports the JD-R assumption that chronic demands contribute to burnout [58]. Importantly, the results suggest that not all demands are equally relevant, as topic-related and client-related demands did not emerge as consistent predictors. This contrasts with earlier research highlighting the emotional burden of client-related issues [21,22,24] and suggests that structural aspects of work may be more critical than the content of interactions [43,54].

One explanation is that workers may develop coping strategies for managing difficult topics and client behaviours, whereas structural demands are persistent, less controllable, and inherent to the work, making them more difficult to regulate [22,47]. In addition, repeated exposure to challenging content may lead to adaptation, while structural uncertainties remain unpredictable and cognitively demanding [22,24].

Contrary to expectations derived from the JD-R model, job resources demonstrated a limited role. Although negatively correlated with burnout, they showed minimal predictive power and did not substantially contribute beyond job demands in most models. This finding only partially supports the motivational pathway of the JD-R model and may reflect the dominance of high-intensity demands, which resources may not fully offset [45,46,59].

An exception was observed for mental distance, where resources contributed significantly. Organizational resources were negatively associated with mental distance, consistent with their protective role [21,23], whereas personal resources were positively associated. This unexpected finding may reflect the use of adaptive coping strategies, such as emotional detachment, which can reduce short-term strain but may also manifest as increased distance from work [47]. Alternatively, it may indicate a more complex interplay between demands and resources in this context.

### 4.1. Theoretical, Methodological, and Practical Implications

The present study addresses several limitations in helpline research, which has been predominantly client-oriented, fragmented, and theoretically under-integrated [3,12,21,22]. In particular, prior studies have rarely differentiated among types of job demands, despite evidence that helpline work involves distinct stressors related to client topics, behaviours, and the service’s structural characteristics. Furthermore, the predominance of volunteer-based samples limits the applicability of findings to professionalized contexts, such as those in Slovakia and the Czech Republic.

By addressing these gaps, the study contributes to the JD-R literature through a context-sensitive application of the model. Differentiating between topic-, client-, and service-related demands provides a more precise operationalization of work characteristics, while the findings challenge the assumption of a uniformly protective role of job resources. Instead, the effectiveness of resources appears context-dependent and varies across burnout dimensions, highlighting the need for a more nuanced application of the JD-R model in high-intensity and structurally constrained environments [46,59].

Methodologically, the study demonstrates the value of context-specific measurement tools. The use of an author-developed instrument enabled a more detailed assessment of helpline-specific demands and resources, which may not be fully captured by standard instruments. In addition, the findings support conceptualizing burnout as a multidimensional construct, as the different dimensions showed distinct patterns of association.

From a practical perspective, the results highlight the potential importance of addressing structural aspects of helpline work in burnout prevention. At both organizational and system levels, improving working conditions may represent an important component of service quality and sustainability within mental health care systems. Given the prominent role of service-related demands, interventions could focus on workload management, adequate staffing, shift organization, reducing uncertainty in interactions, and providing clearer guidelines for ethical decision making [34,43]. Regular supervision and peer consultation may also be beneficial, particularly following high-risk or emotionally demanding contacts. Particular attention could be given to training in text-based communication, where the absence of non-verbal cues may increase interpretative demands [15,28], as well as to emotional regulation and boundary management in anonymous and digitally mediated interactions.

The findings further highlight the potential role of organizational resources, especially supervision and team support, in reducing mental distance and maintaining engagement. Creating supportive team climates and opportunities for reflective practice may further help sustain long-term worker engagement. In contrast, the role of personal resources appears more complex, suggesting that training aimed at strengthening coping strategies may also need to consider potential unintended effects, such as emotional disengagement [53]. The findings also suggest the potential value of integrating occupational well-being into organizational standards and quality assurance frameworks to support accessible, sustainable helpline services.

The findings may also have broader implications for the institutional recognition of helpline services within mental health care systems. In contexts such as Slovakia and the Czech Republic, where anonymous psychosocial helpline services are increasingly integrated into mental health support yet remain only partially formalized at the system level, the development of clearer organizational and professional standards may improve both worker well-being and service sustainability. This could include greater emphasis on supervision standards, workforce protection, training opportunities, and quality assurance procedures for remote psychosocial support services.

### 4.2. Limitations and Future Directions

Several limitations should be acknowledged.

First, the cross-sectional design limits causal interpretation. Future research could employ longitudinal designs to examine the temporal dynamics between job demands, resources, and burnout, including cumulative and lagged effects.

Second, reliance on self-report measures may introduce common-method bias and social desirability effects. Future studies could incorporate multi-source data, such as supervisor ratings, peer evaluations, or objective indicators (e.g., workload metrics, number of contacts, shift duration), to provide a more comprehensive assessment.

Third, the relatively small sample, recruited through snowball sampling, limits generalizability but reflects the hard-to-reach nature of helpline workers. This is consistent with prior research in the field, which often relies on small samples (e.g., [56,65,66,67], as cited in Willems et al. [3]). Future research could benefit from including larger and more diverse samples across organizations and countries.

Fourth, the present study provides preliminary evidence primarily regarding the internal consistency and content relevance of the author-developed instrument rather than full psychometric validation. Given the exploratory pilot nature of the study and the relatively small sample size, more advanced psychometric procedures, such as exploratory or confirmatory factor analyses and formal assessment of convergent and discriminant validity, were not considered sufficiently robust within the present dataset. Although formal convergent and discriminant validity analyses were not conducted, the observed pattern of associations provided preliminary evidence consistent with the JD-R framework’s theoretical assumptions. Therefore, the findings related to the author-developed scales should be interpreted as preliminary and context-specific. Future research with larger, more diverse samples is needed to further examine the factorial structure, construct validity, and broader psychometric properties of the instrument.

Fifth, the present study also did not account for several potentially relevant work-related variables, such as workload, length of professional experience, employment type, or communication modality. These factors may be associated with both the perception of job demands and burnout outcomes and may therefore contribute to variability in the observed associations. Given the relatively small sample size and the exploratory, pilot nature of the study, these variables were not included in the regression models to avoid overfitting and potentially unstable estimates. Future research on larger samples could further explore the role of these variables, including their potential moderating or confounding associations within the JD-R framework.

Finally, the study focused on paid helpline workers in Slovakia and the Czech Republic. Future studies could compare paid and volunteer-based systems, as differences in motivation, workload, and organizational structure may influence burnout processes.

Beyond these limitations, the findings suggest several directions for future research.

First, further investigation of service-related demands may help clarify the role of structural stressors such as uncertainty, lack of feedback, anonymity, and absence of non-verbal cues, including potential differences across communication modalities.

Second, future research could examine interaction effects within the JD-R framework, particularly whether resources moderate the relationship between specific demands and burnout. The present findings suggest that resources may not function uniformly as buffers.

Third, future research could further examine work-related characteristics such as workload, length of professional experience, employment type, and communication modality, as these variables may be associated with both perceived job demands and burnout outcomes. In particular, larger studies could explore their potential moderating or confounding relationships within the JD-R framework and examine whether patterns of burnout differ across specific organizational and communication contexts of helpline work.

Fourth, the unexpected positive association between personal resources and mental distance warrants further investigation, particularly regarding whether certain coping strategies (e.g., emotional detachment) simultaneously reduce strain while increasing disengagement.

Finally, future studies could further consider the multidimensional nature of burnout and explore how emerging technologies, such as AI-assisted communication or hybrid service models, may influence job demands and resources in helpline contexts.

## 5. Conclusions

The present exploratory pilot study examined burnout among helpline workers within the JD-R framework, with a particular focus on differentiating types of job demands in anonymous helpline services. The findings suggest that service-related demands may play a more prominent role in burnout than topic- or client-related demands, highlighting the importance of structural characteristics of helpline work, such as anonymity, emotional intensity, uncertainty, and time pressure. Job resources showed a more limited, context-dependent association with burnout, suggesting the need for a context-sensitive application of the JD-R model in digitally mediated helping environments. Overall, the study contributes to a more nuanced understanding of burnout in helpline work and underscores the importance of supporting helpline workers’ well-being as part of sustainable, accessible mental health support systems.

## Figures and Tables

**Table 1:** Sample characteristics (sociodemographic and work-related variables).

		N	%
Gender			
	*Men*	10	14
	*Women*	63	86
Contract			
	*Full-time*	24	33
	*Part-time*	13	18
	*Casual work contract*	36	49
In-person counselling			
	*Yes*	23	32
	*No*	50	68
Professional orientation			
	*Psychology*	43	59
	*Social work*	22	30
	*Other*	8	11
Number of communication channels			
	*One*	15	20
	*Two*	31	43
	*Three*	27	37
Work location			
	*Home office*	11	15
	*On-site*	52	71
	*Combination*	10	14

**Table 2:** Descriptive statistics for study variables.

		M	SD	Me	Skew	Kurt	Shapiro–Wilk W	*p*
Burnout		1.86	0.53	1.83	3.04	16.70	0.77	<0.001
	*Exhaustion*	2.38	0.74	2.33	0.83	1.31	0.94	0.002
	*Mental distance*	1.63	0.68	1.67	2.51	9.24	0.75	<0.001
	*Cognitive impairment*	1.89	0.64	2.00	1.94	7.50	0.83	<0.001
	*Emotional impairment*	1.53	0.63	1.33	2.72	12.05	0.73	<0.001
Demands		2.99	0.49	2.93	0.88	2.52	0.96	0.011
	*Topic-related*	3.25	0.81	3.25	0.12	−0.33	0.99	0544
	*Client-related*	3.13	0.75	3.20	0.11	0.12	0.99	0.653
	*Service-related*	2.85	0.49	2.85	0.97	2.71	0.95	0.005
Resources		4.24	0.44	4.25	−0.81	1.48	0.96	0.011
	*Personal*	4.14	0.59	4.25	−0.78	0.43	0.94	0.003
	*Organizational*	4.31	0.49	4.42	−1.09	1.23	0.92	<0.001

M = mean; SD = standard deviation; Skew = skewness; Kurt = kurtosis; W = Shapiro–Wilk’s test.

**Table 3:** Spearman correlations between study variables.

	BO	EX	MD	CI	EI	D	TR	CR	SR	R	P	O
Burnout	-											
*Exhaustion*	0.78 ***	-										
*Mental distance*	0.69 ***	0.46 ***	-									
*Cognitive impairment*	0.54 ***	0.19	0.25 *	-								
*Emotional impairment*	0.65 ***	0.33 **	0.37 **	0.27 *	-							
Demands	0.41 ***	0.42 ***	0.21	0.12	0.34 ***	-						
*Topic-related*	0.13	0.14	0.05	−0.04	0.14	0.72 ***	-					
*Client-related*	0.21	0.27 *	0.24 *	−0.05	0.16	0.76 ***	0.55 ***	-				
*Service-related*	0.55 ***	0.57 ***	0.27 *	0.23 *	0.44 ***	0.78 ***	0.30 ***	0.37 ***	-			
Resources	−0.30 **	−0.19	−0.23 *	−0.15	−0.19	−0.11	0.08	−0.01	−0.18	-		
*Personal*	−0.25 **	−0.16	−0.11	−0.17	−0.20	−0.22	−0.11	0.01	−0.29 *	0.79 ***	-	
*Organizational*	−0.20	−0.09	−0.27 *	−0.08	−0.11	0.02	0.15	0.05	−0.02	0.84 ***	0.43 ***	-

BO = burnout; EX = exhaustion; MD = mental distance; CI = cognitive impairment; EI = emotional impairment; D = demands; TR = topic-related; CR = client-related; SR = service-related; R = resources; P = personal; O = organizational; * *p* < 0.05; ** *p* < 0.01; *** *p* < 0.001.

**Table 4:** Hierarchical regression analysis—overall burnout.

		Model 1				Model 2			
		b	SE	β	*p*	b	SE	β	*p*
DEMANDS	*Topic-related*	−0.16	0.11	−0.16	0.150	−0.12	0.11	−0.12	0.286
	*Client-related*	0.13	0.10	0.16	0.178	0.11	0.10	0.13	0.276
	*Service-related*	0.34	0.05	0.68	<0.001	0.35	0.05	0.70	<0.001
RESOURCES	*Personal*					0.15	0.13	0.11	0.258
	*Organizational*					−0.14	0.11	−0.13	0.197
	R^2^	0.48 ***				0.50 ***			
	F	21.59				13.40			
	ΔR^2^					0.02			

*** *p* < 0.001.

**Table 5:** Hierarchical regression analysis—exhaustion.

		Model 1				Model 2			
		b	SE	β	*p*	b	SE	β	*p*
DEMANDS	*Topic-related*	−0.04	0.04	−0.12	0.327	−0.04	0.04	−0.11	0.397
	*Client-related*	0.04	0.04	0.13	0.296	0.04	0.04	0.12	0.344
	*Service-related*	0.10	0.02	0.59	<0.001	0.10	0.02	0.60	<0.001
RESOURCES	*Personal*					0.02	0.05	0.05	0.674
	*Organizational*					−0.01	0.04	−0.04	0.743
	R^2^	0.37 ***				0.37 ***			
	F	13.36				7.85			
	ΔR^2^					0.00			

*** *p* < 0.001.

**Table 6:** Hierarchical regression analysis—mental distance.

		Model 1				Model 2			
		b	SE	β	*p*	b	SE	β	*p*
DEMANDS	*Topic-related*	−0.10	0.04	−0.30	0.025	−0.07	0.04	−0.21	0.110
	*Client-related*	0.09	0.04	0.34	0.015	0.07	0.04	0.27	0.043
	*Service-related*	0.07	0.02	0.43	<0.001	0.08	0.02	0.48	<0.001
RESOURCES	*Personal*					0.11	0.05	0.26	0.021
	*Organizational*					−0.11	0.04	−0.31	0.006
	R^2^	0.29 ***				0.38 ***			
	F	9.18				8.06			
	ΔR^2^					0.09 *			

* *p* < 0.05; *** *p* < 0.001.

**Table 7:** Hierarchical regression analysis—cognitive impairment.

		Model 1				Model 2			
		b	SE	β	*p*	b	SE	β	*p*
DEMANDS	*Topic-related*	−0.02	0.04	−0.06	0.647	−0.02	0.04	−0.07	0.636
	*Client-related*	−0.01	0.04	−0.05	0.741	−0.01	0.04	−0.04	0.767
	*Service-related*	0.08	0.02	0.52	<0.001	0.08	0.02	0.52	<0.001
RESOURCES	*Personal*					0.00	0.05	−0.01	−0.01
	*Organizational*					0.01	0.04	0.02	0.02
	R^2^	0.23 ***				0.23 **			
	F	6.89				4.02			
	ΔR^2^					0.00			

** *p* < 0.01; *** *p* < 0.001.

**Table 8:** Hierarchical regression analysis—emotional impairment.

		Model 1				Model 2			
		b	SE	β	*p*	b	SE	β	*p*
DEMANDS	*Topic-related*	−0.00	0.04	−0.01	0.906	0.00	0.04	0.01	0.964
	*Client-related*	0.01	0.03	0.05	0.683	0.01	0.03	0.04	0.776
	*Service-related*	0.09	0.02	0.60	<0.001	0.09	0.02	0.61	<0.001
RESOURCES	*Personal*					0.02	0.04	0.05	0.648
	*Organizational*					−0.02	0.04	−0.07	0.519
	R^2^	0.36 ***				0.34 ***			
	F	14.23				8.44			
	ΔR^2^					0.00			

*** *p* < 0.001.

## Data Availability

The data supporting the findings of this study are available from the corresponding author upon reasonable request. The data are not publicly available due to ethical considerations related to the protection of participant anonymity.
